# Two new plumage mutations in the Japanese quail: "curly" feather and "rusty" plumage

**DOI:** 10.1186/1471-2156-6-14

**Published:** 2005-03-11

**Authors:** Francis Minvielle, David Gourichon, Chantal Moussu

**Affiliations:** 1 Génétique et Diversité Animales, Institut National de la Recherche Agronomique, Centre de Jouy, 78352 Jouy-en-Josas, France; 2 Unité Expérimentale de Génétique Factorielle Avicole, Institut National de la Recherche Agronomique, Centre de Tours, 37380 Nouzilly, France

## Abstract

**Background:**

The genetics of plumage of Japanese quail is of interest both from a biological standpoint, for comparative studies between avian species, and from a zootechnical standpoint, for identifying commercial selection lines or crosses. There are only few plumage mutations reported in quail, and the present work describes a new color variant "rusty" and a new feather structure "curly", and their heredity from an F1 and F2 segregation experiment.

**Results:**

Curly feathers result from abnormal early growth caused by transient joining of follicle walls of adjacent feathers around 10 days of age, but the expression of the trait is variable. Rusty plumage color results from the replacement of the wild-type plumage pattern on the tip of the feather by a reddish coloration, but the pigmentation of the bottom part of the feather is not affected. Two lines breeding true for the curly or the rusty phenotype were developed. Both characters are determined by autosomal recessive mutations which are independent. The curly mutation has also a positive effect on body weight at 5 weeks of age.

**Conclusion:**

The curly line is a new model which may be used for further work on the growth of the feather, and the rusty mutation is a new addition to the panel of plumage mutations available for comparative studies in poultry, and more generally among avian species.

## Background

Japanese quail is both a model animal in biology and a bird used for meat and egg production under a large variety of settings [1]. In the recent past, a special attention was given to the study of its plumage, and several major genes have been described [2]. Since the last compilation of plumage mutations of Japanese quail [2], new loci were described [e.g. [3]], linkage and epistasis relationships were explored [4,5], and some genes were recently mapped [6]. This knowledge has already been put to use for running comparisons between chicken and quail based on plumage genetics [7,8], and for tagging commercial quail lines with a visible plumage trait, like the "fawn" mutation [9], or with an auto-sexing mutation like the "roux" gene [10]. Interestingly, some of the mutations described in quail, like the sex-linked "roux" and the lethal "yellow" mutations still have no known homologues in the chicken. Moreover, the fact that some plumage colors, like "lavender", are common to several avian species [5] is an added incentive to enrich the panel of characterised Japanese quail mutations as potential tools for comparative studies among bird species.

In the present work, a new feather structure phenotype (curly) and a new plumage color (rusty) were described, and their mode of inheritance and linkage were studied in two successive generations (F1 and F2) from an F0 made of eight quail with curly feathering and eight birds with rusty plumage. Growth of the F2 quail was also monitored and compared according to their phenotype for the two plumage mutations.

## Results and discussion

### Phenotypic description

**Figure 1:**
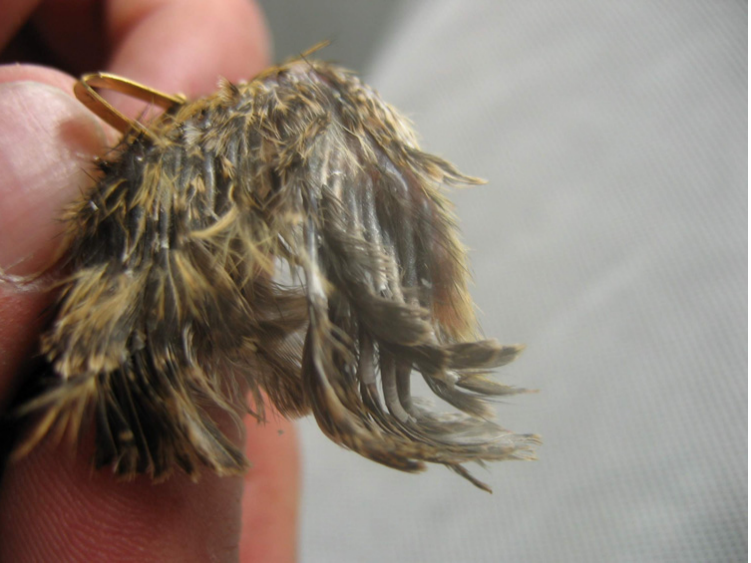
**Wing of a 10 day Japanese quail with the curly phenotype. **In curly quail, the calamus of the adjacent growing wing feathers are not independent from one another, but they are connected through the follicle walls which appear to be joined together. This phenomenon which lasts one to two weeks is best observed around 10 days of age. It hinders the normal growth of the feathers, thereby inducing the permanent curly structure of the feathers.

**Figure 2:**
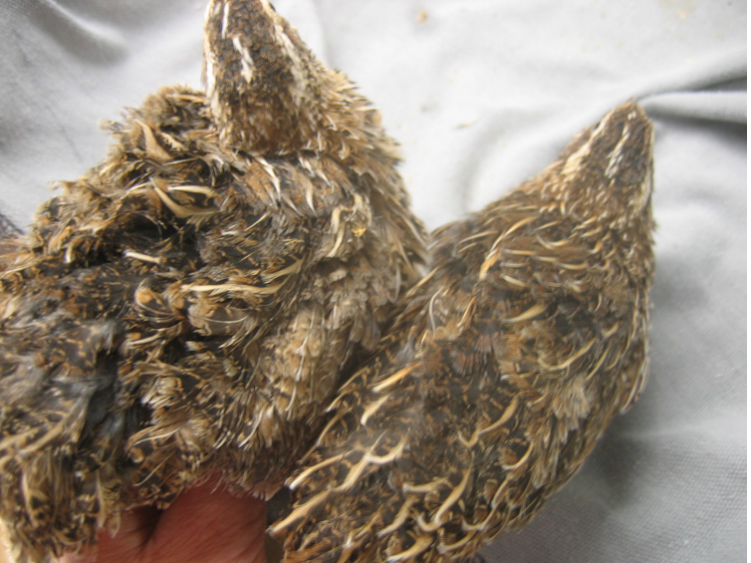
**Japanese quail with the curly and the normal feather structures. **The adult curly quail is on the left. Its plumage has an overall fluffier look when compared to the normal quail on the right, but the intensity of the curliness varies between curly quail, as the penetrance of this autosomal recessive mutation is not complete.

The quail line with the curly feathers was developed in 2001 from group mating 6 founding (G0) curly quail. Starting in G3, only quail for which the curly phenotype observed at 10 days of age was expressed most strongly were kept for breeding. No differential survival was observed in curly quail after hatching, but the hatching rate in the fixed line at G5 under pool mating was only 34%, mainly because some females did not get mated under this type of mating. In curly chicks, the calamus of the growing wing feathers are not independent from one another, but they are connected through the follicle walls which appear to be joined together. This phenomenon is best observed around 10 days of age and is associated with the curly growth (Figure 1). The expression of the trait is variable, however, and the penetrance of the curly mutation appears to be incomplete. The difference between normal and curly adults (Figure 2) is not as marked as for other feather structure mutations, like porcupine [11], for example. Following the gene nomenclature proposed for the chicken [12], the locus for this new mutation was named CU, and the symbols of the allele responsible for the curly mutation and of the normal allele at this locus were CU*C and CU*N, respectively. In all instances, inheritance of the trait was similar for both sexes, and heterozygotes CU*C/CU*N had normal plumage structure, indicating that the locus CU was autosomal and the mutation was recessive.

**Figure 3:**
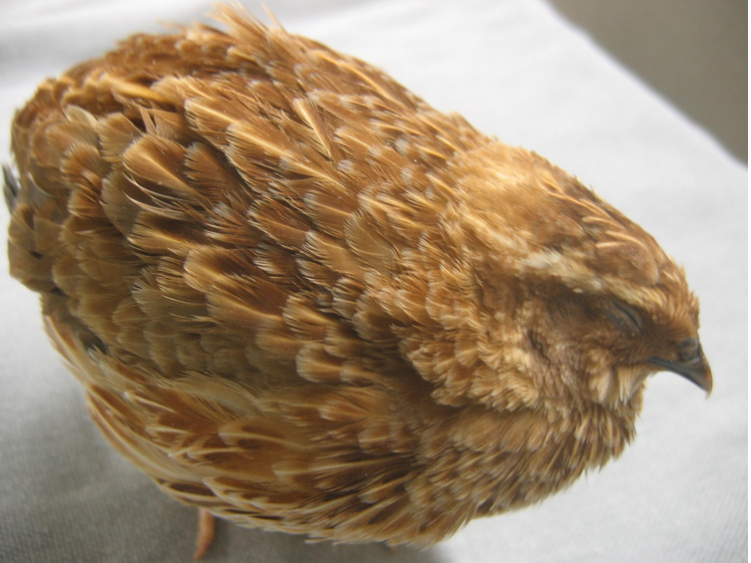
**Japanese quail with the rusty plumage color. **Quail with the rusty phenotype are homozygous for an autosomal recessive mutation.

**Figure 4:**
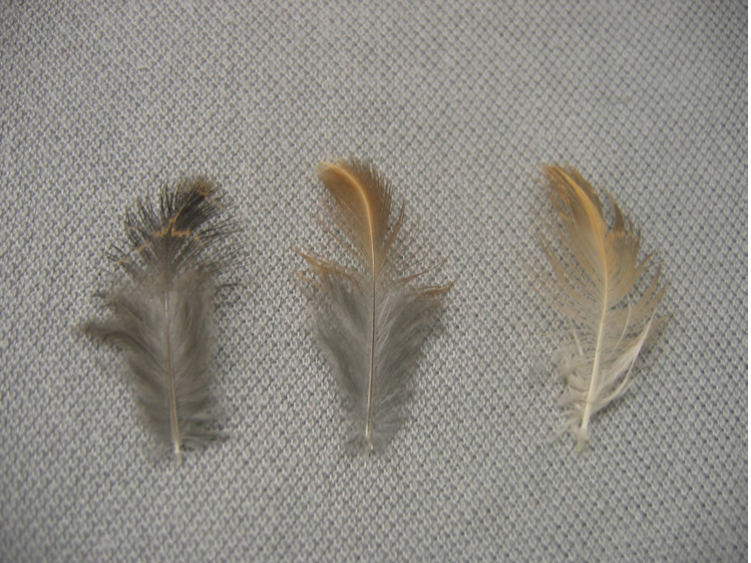
**Comparison of colors of feathers from Japanese quail. **On the left, the contour feather of a wild-type Japanese quail shows the usual brownish colored tip with a transversal lighter stripe and slaty colored barbs at the bottom. On the center, the feather of a quail with the rusty phenotype has a different, rusty colored, tip, but keeps the same slaty colored barbs than the wild-type bird at the bottom. On the right, the feather of a roux quail (caused by a sex-linked recessive mutation) shows a diluted color, paler than rusty, over its whole length.

The quail line with the rusty plumage color was started in 2000 from a founding base (G0) made of a single rusty female bred to a wild-type male from another origin. It was followed by sib mating of the all wild-type G1 quail to produce G2 and G3 pedigreed progeny and selection of the few rusty G4 birds. Survival rate to sexual maturity of the rusty descendance of the early generations was poor, and reproductive performance was low (hatched/incubated = 31%) in the fixed line under pool mating at G6, because of the mating system but also of inbreeding derived from having a single rusty ancestor. The plumage of the mutant chicks was rusty, but the down underneath retained the usual wild-type dark-slaty color. A similar color pattern was maintained in rusty adults: their contour and flight feathers had dark barbs on the bottom and rusty colored barbs on the top third of their length which produced the overall rusty look (Figure 3). The effect of this mutation appears to be different from the dilution of the pigmentation [5] associated with the roux mutation which affects the whole feather and produces a paler color (Figure 4). The locus for this new mutation was named RU, and the symbols of the allele responsible for the rusty mutation and of the wild-type allele at this locus were RU*R and RU*N, respectively. In all instances, inheritance of the trait was similar for both sexes, and heterozygotes RU*R/RU*N had wild-type plumage color, indicating that the locus RU was autosomal and the mutation was recessive.

### Linkage analysis

**Figure 5:**
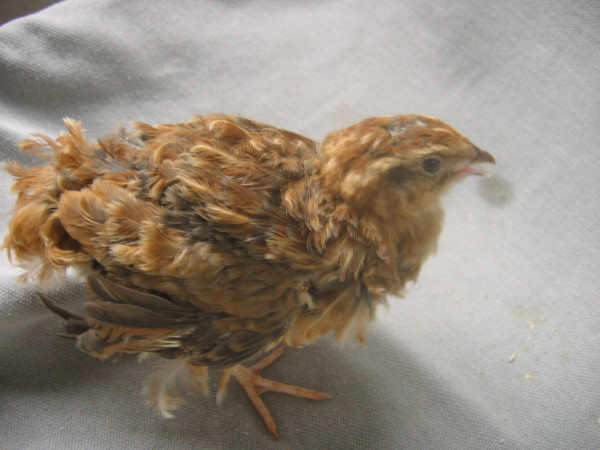
**Japanese quail with the rusty plumage color and the curly feather structure. **This phenotype was the least frequent of the four quail types produced in the F2 between the curly and the rusty lines. These quail are homozygous for the two recessive mutations.

All 68 F1 quail were wild-type birds, confirming that both mutations should be recessive, and not sex-linked. Four phenotypes were obtained in the 531 F2 progeny: 326"wild-type", 95 "rusty", 79 "curly" and 31 "rusty and curly" (Figure 5). The high hatching rate (81%) obtained in the F2 confirms that the much lower value obtained in the two lines fixed for the rusty or the curly mutation might have resulted from inbreeding due to the small number of founders and from the pool mating system used rather than from detrimental reproductive effects directly associated with the two mutations. Observed and expected distributions under two different null hypotheses (A: independent segregation given complete penetrance of the curly mutation, and B: independent segregation given incomplete penetrance of the curly mutation) are shown with the corresponding values of χ^2^_s _(7.5 and 1.9, respectively) in Table 1. None of the two hypotheses could be rejected because the probabilities for these values of χ^2^_s _were high enough (p > 0.05 and p > 0.1, respectively). The maximum likelihood estimation of the penetrance parameter (± SE) was 1-λ = 0.83 (± 0.07) which indicates that 17% of F2 curly quail might have been misclassified as wild-type birds. This result is consistent with the observation that the expression of the curly trait was variable, and it would account for the relative deficit of curly birds in the F2. Overall, however, the segregation of the F2 results fits a simple two-locus Mendelian inheritance of two autosomal recessive and independent mutations.

**Table 1:** Segregation of plumage color and feather structure in the F2 from rusty plumage and curly feather quail lines

Phenotype	Observed (n = 531)	Expected under independent segregation	
		
		And complete penetrance (λ = 0)	And incomplete penetrance (0 < λ < 1)
Wild-type	a = 326	9n/16 = 298.7	9n/16+3λn/16 = 315.6^¥^
Rusty	b = 95	3n/16 = 99.6	3n/16+λn/16 = 105.2
Curly	c = 79	3n/16 = 99.6	3n(1-λ)/16 = 82.7
Rusty and curly	d = 31	n/16 = 33.2	n(1-λ)/16 = 27.6
Goodness of fit		χ^2^_s _= 7.5^£^	χ^2^_s _= 1.9^$^

^¥^: calculated using 1-λ = 0.83

^£^:0.10 > p > 0.05.

^$^:0.5 > p > 0.1

### Growth

At hatching, none of the plumage mutations were associated with differences of body weight (Table 2), but after two weeks quail with curly feathers had become significantly heavier than wild-type ones, and the difference reached 3.4% (p < 0.001) of the average body weight at 35 days of age (Table 2). On the opposite, quail with rusty plumage were 2.3 % lighter (p < 0.05) than wild-type ones. The mean body weight at 5 weeks of age was 176.8, 170.5, 181.0 and 182.0 g, respectively for the "wild-type", "rusty", "curly" and "rusty and curly" quail. The fact that both "curly" and "rusty and curly" birds had a similar high body weight, whereas "rusty" quail were the smallest ones is an indication that the effect on growth of the "curly" mutation might be epistatic over that of the "rusty" mutation. The association of plumage color mutations with lower growth have been previously reported in albino [13] and roux [10] quail, with respectively 9 and 3% lower body weight at a similar age, but no mutation with a favorable effect had been found so far. A large scale experiment focused on the associated effects of the curly mutation on growth is needed, however, to confirm and extend the present results.

**Table 2:** Analyses of variance of body weight of the F2 from rusty plumage and curly feather quail lines

Age	At hatch	1 week	3 weeks	5 weeks
Sample size^1^	348	348	346	347
Mean body weight (g) (SD)	8.1 (0.8)	27.6 (4.5)	105.7 (12.5)	176.7 (17.0)
R^2^	0.82	0.45	0.47	0.50
Significance of main effects				
Hatch	**	ns	***	***
Family	***	***	***	***
Sex	**	ns	ns	***
Feather structure	ns	ns	**	***
Plumage color	ns	***	**	*
Contrast (g)				
"curly" – "wild-type"	-0.08^ns^	0.8^ns^	3.8**	6.1***
"rusty" – "wild-type"	0.04^ns^	-2.1***	-3.6**	-4.1*

^1^: The quail sample was made up of 215 wild-type, 59 curly, 58 rusty, and 16 curly and rusty quail from 17 full-sib families and born in two successive hatches. *: p < 0.05; **: p < 0.01; ***: p < 0.001; ^ns^: not significant.

## Conclusion

The two new "curly" and "rusty" mutations will enrich the small number of plumage mutations already available in Japanese quail for studying the genetics and the biology of feathers, a field of research with many perspectives [14]. They may have also some interest from a zootechnical standpoint to tag commercial lines, and, if the positive effect of the "curly" mutation was confirmed, this gene might be worth introgressing in parental meat quail lines.

## Methods

### Birds

The two mutations originated in the experimental quail population maintained and selected on behavioral traits at the INRA Station de recherches avicoles in Nouzilly, France. After two quail lines were established by fixing separately the curly and the rusty phenotypes, eight reciprocal single pair matings (three "rusty × curly" and five "curly × rusty") were set up with G5 rusty and G4 curly quail from the two pure lines to produce the F1. Then, 531 F2 birds were produced in three consecutive hatches from 17 single pair matings of F1 birds. Sib-mating was avoided, and the hatching rate across all pair matings and hatches was 81%. All chicks were pedigreed, and they were phenotyped for plumage color at hatching and for feather structure at 10 days of age. F2 quail from the first two hatches were raised in two group pens (one per hatch) with free access to ad libitum commercial feed and drinking water, and they were weighed weekly until 5 weeks of age.

### Genes

Gene nomenclature used in this paper followed recommendations published for chicken genes [12], with a two-part symbol: "locus abbreviation"*"allele abbreviation/locus", and "locus abbreviation*N" as the symbol for the wild-type allele.

### Statistical analyses

Analysis of the segregation in the F2 to test for linkage was carried out using maximum likelihood methodology and the χ^2 ^test [15]. Penetrance and its standard error were estimated as: 1-λ = (3c/(a+c)) + d(b+d) and SE = (9ac/(a+c)^3^) + bd/(b+d)^3^, derived for misclassification of phenotypes due to "partial manifestation" [16]. In the formulae, a, b, c and d are the numbers of observations in the four phenotypic classes described in Table 1.

Five-way analyses of variance of individual body weights (BW) between hatching and 5 weeks of age were carried out by the GLM procedure [17] for the 348 quail hatched alive in the first two hatches, using the following linear model: BW=(overall mean) + hatch + family + sex + (feather structure phenotype) + (plumage color phenotype) + error. The number of classes for the five main effects were respectively, 2, 17, 2, 2 and 2. Contrasts between least-squared means for each mutant phenotype and for the wild-type quail were estimated from the analyses of variance, using data adjusted for systematic effects of hatch, family and sex.

## Authors' contributions

FM coordinated the study and wrote the paper, DG and CM participated in the design of the study and carried out the the data collection.
